# Pelviscopic Compared to Laparotomic and Vaginal Intrafascial Hysterectomy

**DOI:** 10.1155/DTE.3.231

**Published:** 1997

**Authors:** L. Mettler, N. Lutzewitsch

**Affiliations:** Department of Obstetrics and Gynecology of the Christian-Albrechts-University Kiel Michaelisstraße 16 Kiel D-24105 Germany

## Abstract

Between 1993 and 1994, 368 women underwent hysterectomies for benign disorders at the University of Kiel. Of these, 58.7% were performed either by pelviscopic or by laparotomy Classic Intrafascial Supracervical Hysterectomy (CISH). Of the remaining, 14.8% were performed by abdominal hysterectomy, 13.6% by Intrafascial Vaginal Hysterectomy (IVH), 12.2% by Vaginal Hysterectomy (VH), and only 0.05% by Laparoscopic Assisted Vaginal Hysterectomy (LAVH). Comparative data of these six surgical techniques concerning patients characteristics, indications for operation, histological features, blood loss, operating time, hospital stay, uterine weights and postoperatively used analgesics are described.


*Diagnostic and Therapeutic Endoscopy*, 1997, Vol. 3, pp. 231-239
Reprints available directly from the publisher
Photocopying permitted by license only

© 1997 OPA (Overseas Publishers Association)
Amsterdam B.V. Published in The Netherlands
by Harwood Academic Publishers
Printed in Singapore

### Pelviscopic Compared to Laparotomic and Vaginal Intrafascial Hysterectomy

L. METTLER* and N. LUTZEWITSCH

*Department of Obstetrics and Gynecology of the Christian-Albrechts-University Kiel, Michaelisstraße 16, D-24105 Kiel, Germany*

*(Received 29 January 1996; in final form 20 September 1996)*

**Between 1993 and 1994, 368 women underwent hysterectomies for benign disorders at the University of Kiel. Of these, 58.7% were performed either by pelviscopic or by laparotomy Classic Intrafascial Supracervical Hysterectomy (CISH). Of the remaining, 14.8% were performed by abdominal hysterectomy, 13.6% by Intrafascial Vaginal Hysterectomy (IVH), 12.2% by Vaginal Hysterectomy (VH), and only 0.05% by Laparoscopic Assisted Vaginal Hysterectomy (LAVH). Comparative data of these six surgical techniques concerning patients characteristics, indications for operation, histological features, blood loss, operating time, hospital stay, uterine weights and postoperatively used analgesics are described.**

*Keywords:* Subtotal hysterectomies, CISH

### INTRODUCTION

The first carefully described abdominal supracervical hysterectomy was performed by Wilhelm Alexander Freund in 1878 and it was the leading technique for over 80 years [1]. In 1963 Tervilä [2] described the danger of cervical cancer to be 0.3–1.9% following supracervical hysterectomy. From 1950 onwards hysterectomy was almost only performed as total hysterectomy. Since the 1990 interest in supracervical hysterectomy has been re-established because of the introduction of the Classic Intrafascial Supracervical Hysterectomy pelviscopic and laparotomy techniques [3–9]. Previously, in 1984 Semm described the separation of the adnexa pelviscopically to facilitate vaginal hysterectomy, it was then called vaginal hysterectomy with pelviscopic assistance [10]. In 1989 Reich successfully advanced the procedure by including the ligation of the uterine artery and removal of the uterus via colpotomy[11].

During the past few years, new techniques have completed the vaginal and abdominal techniques of

*Correspondence: Prof. L. Mettler, Department of Obstetrics and Gynecology of the Christian-Albrechts-University Kiel, Michaelisstraße 16, D-24105 Kiel, Germany. Tel.: 049 0431 597 2100; Fax: 049 0431 597 2149.

Table I The different techniques of hysterectomy applied in 36 patients (1993–1994)

| | Number of patients |
|---|---|
| Vaginal hysterectomy (VH) | 46 |
| Abdominal hysterectomy | 54 |
| Laparotomy CISH procedure (Lp CISH) | 103 |
| Pelviscopy CISH procedure (Pelv. CISH) | 113 |
| Intrafascial Vaginal Hysterectomy (IVH) | 50 |
| Laparoscopically assisted Vaginal Hysterectomy (LAVH) | 2 |
| n | 368 |

hysterectomy. The first CISH was successfully performed by Semm in Kiel, Germany on September 7, 1991. It combines the advantages of traditional supracervical hysterectomy, including shorter operative time and preservation of major parts of the pelvic floor, with prevention of cervial cancers by coring the inner cervix with resection of the transformation zone. From the CISH procedure the Intrafascial Vaginal Hysterectomy (IVH) was developed as a new minimal invasive technique. Prior to 1991 all hysterectomies for benign or malign indications were performed transabdominally or transvaginally.

### MATERIALS AND METHODS

Between January 1993 and December 1994, 366 hysterectomies for benign indications were performed in the Department of Obstetrics and Gynaecology, Kiel University on women with benign indications for hysterectomy (46 vaginal hysterectomies, 54 abdominal hysterectomies, 103 laparotomy CISH, 113 pelviscopic CISH operations, 50 IVH and 2 LAVH), Table I.

Patients were assigned within a benign indication for hysterectomy to an individual group based on the size of the uterus, patient's complaints and the experience of the surgeon. Pelviscopic hysterectomy was not considered if the uterus was larger than 12 weeks gestation. Cervical preservation was only suggested in cases with no previous or present cervical pathology.

The operating time (OP-time) was calculated from the entrance of the anaesthetised patient into the operating theatre (OT) till the end of surgery. The patient's preparation in the operating theatre needed an average of 15 min, which should be subtracted from the OP-time.

All data were analysed by the repeated-measures analysis of variance, when appropriate, $p < 0.01$ was considered statistically significant.

### THE TECHNICAL DETAILS

### Pelviscopic CISH

The cervix is grasped at 3 and 9 o'clock with two tenaculae, and the cervical canal is dilated up to Hegar 6. To minimise intraoperative blood loss 10–20 ml POR-8 (synthetic vasopressin derivative-Sandoz®) in 0.05% dilution are injected into the cervix. The perforation rod is introduced transcervically up and through the fundus uteri under pelviscopic control. To core out the transcervical-transuterine cylinder we use a 15, 20 or 24 mm calibrated uterine resection tool (CURT). The setting of the tool depends upon the diameter of the cervix, which had previously been measured with a sonographic examination. The remaining cervix is endocoagulated at 100°C to 120°C. The bladder is separated from the cervix and vagina by aquadissection. Adnexal dissection either from the uterus or from the pelvic wall is carried out bilaterally with sutures or staples to the level of the cardinal ligaments. Three Roeder loops are placed around the cervix. The uterine body is separated from the cervix with scissors. The cervical stump is suspended to the round ligaments and peritonealized. The uterus is morcellated and extracted by using the SEMM = Serrated Edged Macro Morcellator.

### CISH by Laparotomy

After performing abdominal subtotal hysterectomy, the perforation rod is introduced through the cervical stump and the cervical canal is cored out with the CURT and the remaining cervical tissue is coagulated as above.

### Intrafascial Vaginal Hysterectomy (IVH)

After having punched out the cervical tissue with the CURT, vaginal subtotal hysterectomy is then performed with the Stoeckel [12] technique. Using only an anterior colpotomy incision, the uterovesical pouch is opened, delivering the uterus anteriorly through the vaginal incision and thereby excluding a dissection of both cardinal and sacrouterine ligaments with the cervix in place.

### Vaginal hysterectomy.

The Stoeckel technique with resection of the uterus through an anterior colpotomy is performed without coring the cervical canal.

### Total abdominal hysterectomy

Total abdominal hysterectomy is performed as described by Freund and Stoeckel.

### Laparoscopic assisted vaginal hysterectomy (LAVH)

Hysterectomies are initially started with pelviscopic separation of the adnexea from the uterus or the pelvic wall, and always completed vaginally.

### RESULTS

The different techniques used on the 368 patients are presented in Figure 1a and 1b. No significant difference in mean parity and body-mass index were found between all groups of patients (Fig. 2 and 3).

The mean age of the patients that underwent IVH (47.5 years), abdominal and pelviscopic CISH (47.9 years and 46.9 years) were significantly less than that of vaginal hysterectomy (62 years) and abdominal hysterectomy (55.8 years) (Fig. 4). Table II shows the different indications for hysterectomy.

The decision for the individual surgical procedure was on the basis of thorough history, physical

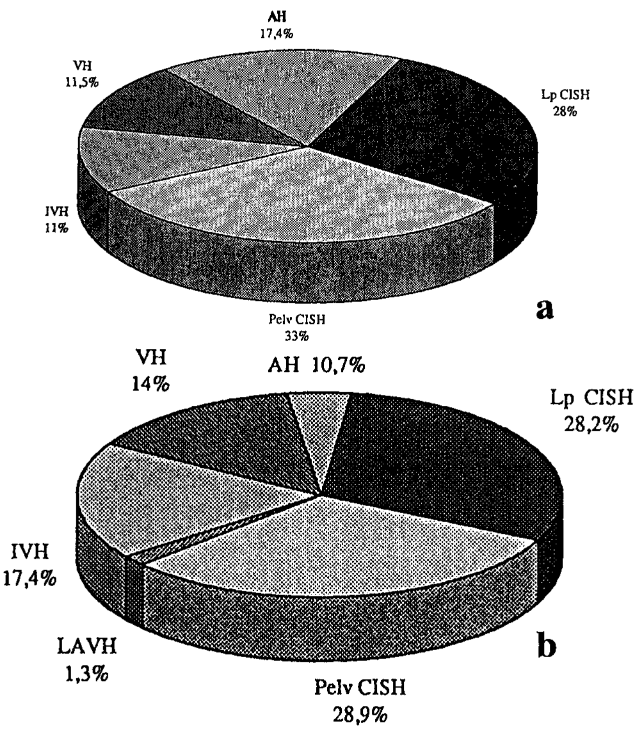

FIGURE 1 Hysterectomies for benign indications (1993) a and (1994) b. Abdominal hysterectomy – AH; Laparotomic Classic Intrafascial Supracervical Hysterectomy – Lp. CISH; Pelviscopic Classic Intrafascial Supracervical Hysterectomy – Pelv. CISH; Vaginal hysterectomy – VH; Intrafascial vaginal hysterectomy– IVH; Laparoscopic Assisted Vaginal Hysterectomy – LAVH.

Table II Indications for hysterectomy (per cent), (n=368, 1993–1994)

| | VH | AH | Abd.CISH | Pelv.CISH | IVH |
|---|---|---|---|---|---|
| Myomas with menstrual abnormalities | 9.09 | 25.58 | 27.72 | 33.96 | 36 |
| Myomas with pressure symtomps | 4.54 | 37.20 | 45.54 | 17.92 | 10 |
| Endometriosis | 0 | 2.3 | 0 | 2.8 | 0 |
| Chronic pelvic pain | 2.27 | 2.3 | 1.9 | 6.6 | 2 |
| Menometrorrhagia and hypermenorrhea | 13.63 | 9.29 | 13.86 | 33.02 | 28 |
| Dysfunctional bleeding refractory to therapy | 9.11 | 11.71 | 7.34 | 3.82 | 2 |
| Prolapse | 61.36 | 11.62 | 3.9 | 1.88 | 22 |
| **per cent** | 100 | 100 | 100 | 100 | 100 |

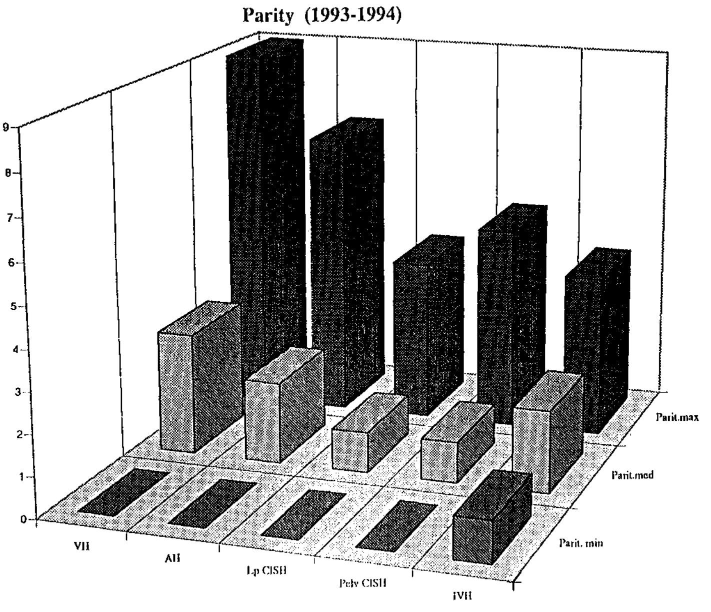

FIGURE 2 Parity of 368 patients who underwent hysterectomy at the Department of Obstetrics and Gynaecology University of Kiel, Germany (1993–1994). Abdominal Hysterectomy – AH; Laparotomic Classic Intrafascial Supracervical Hysterectomy – Lp. CISH; Pelviscopic Classic Intrafascial Supracervical Hysterectomy – Pelv. CISH; Vaginal Hysterectomy – VH; Intrafascial Vaginal Hysterectomy – IVH.

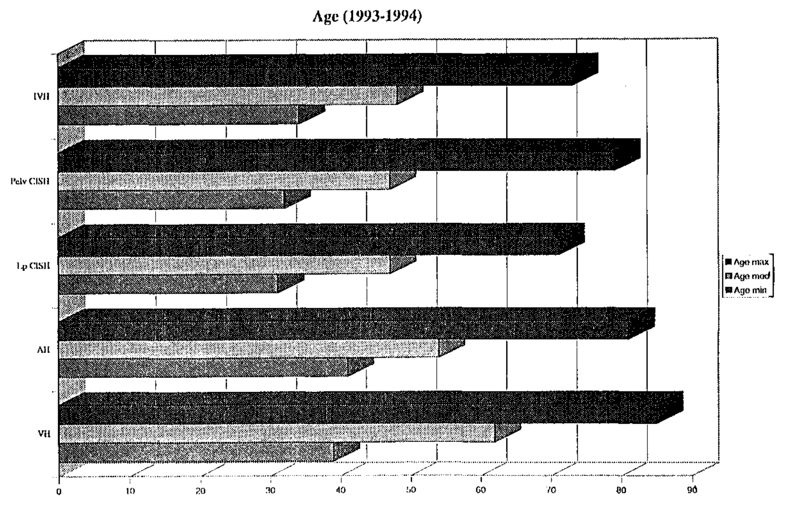

FIGURE 3 Age distribution of 368 patients who underwent hysterectomy at the Department of Obstetrics and Gynaecology University of Kiel (1993–1994). Abdominal Hysterectomy – AH; Laparotomic Classic Intrafascial Supracervical Hysterectomy – Lp. CISH; Pelviscopic Classic Intrafascial Supracervical Hysterectomy – Pelv. CISH; Vaginal Hysterectomy – VH; Intrafascial Vaginal Hysterectomy – IVH.

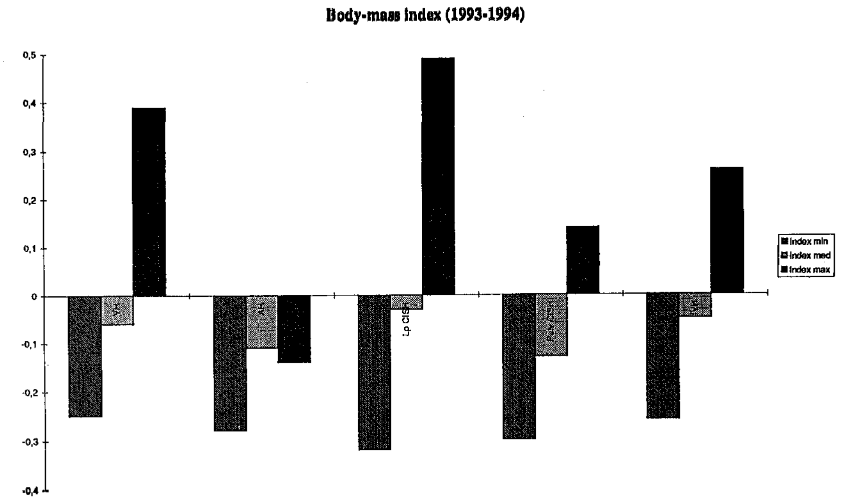

FIGURE 4 Body-mass index of 368 patients who underwent hysterectomy at the Department of Obstetrics and Gynaecology University of Kiel (1993–1994). Abdominal Hysterectomy – AH; Laparotomic Classic Intrafascial Supracervical Hysterectomy – Lp. CISH; Pelviscopic Classic Intrafascial Supracervical Hysterectomy – Pelv. CISH; Vaginal Hysterectomy Intrafascial Vaginal Hysterectomy – IVH; Laparoscopic Assisted Vaginal Hysterectomy.

examination and ultrasonographic evaluation. In 4 cases which started as pelviscopic surgery, an intraoperative conversion to laparotomy was performed. These cases were concluded in the laparotomy CISH group.

The intraoperative blood loss in different procedures is detailed in Fig. 5. A significant difference in blood loss was found between pelviscopic CISH (401 ml), vaginal hysterectomy (387 ml), IVH (364 ml) and laparotomy CISH (575 ml) and abdominal hysterectomy (718 ml).

A comparison of the operating times (Fig. 6) revealed that the pelviscopic procedures took the longest time (158.99 min) compared to vaginal hysterectomy (126.03 min), abdominal hysterectomy (128.68 min), laparotomy CISH (128.68 min) and IVH (60.84 min). 61% of the vaginal hysterectomies were accompanied by anterior and posterior repair.

According to the histopathological findings, the majority of myoma patients were treated by abdominal hysterectomy and by CISH-operations either pelviscopically or through laparotomy. While patients with genital descent were mostly operated using the vaginal hysterectomy or IVH techniques, showed normal histology (Table III).

The uterine size and weight could significantly affect the type of the hysterectomy operation needed. The average uterine weight was 145 g (range from 33 to 360 g). Figure 7 showed that the uterine weights less than 200 g were removed by IVH and pelviscopic CISH operations, while larger size uteri weighing less than 400 g in average were treated by AH and laparotomy CISH operations.

Table III Histological diagnosis of hysterectomy specimen (n=368, 1993–1994).

| | VH | AH | Lp.CISH | Pelv.CISH | IVH |
|---|---|---|---|---|---|
| Leiomyomas | 27.27 | 51.16 | 54.46 | 53.77 | 36 |
| Adenomyosis | 22.72 | 20.93 | 22.77 | 23.59 | 42 |
| Adenomatous hyperplasia | 6.82 | 6.98 | 1.98 | 2.83 | 4 |
| No pathology | 36.36 | 16.28 | 12.87 | 13.20 | 14 |
| Other pathology | 6.83 | 4.65 | 7.92 | 6.61 | 4 |
| per cent | 100 | 100 | 100 | 100 | 100 |

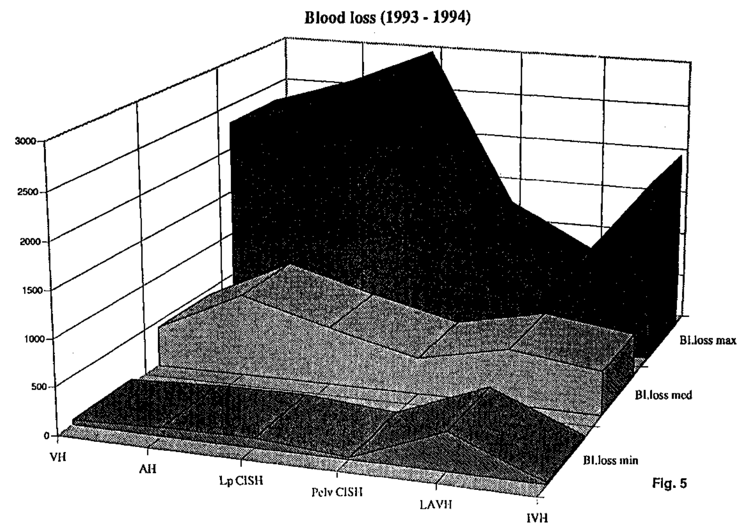

FIGURE 5 Blood loss in 368 hysterectomies of patients who underwent hysterectomy at the Department of Obstetrics and Gynaecology University of Kiel (1993–1994), divided into 6 different techniques: Abdominal hysterectomy – AH; Laparotomic Classic Intrafascial Supracervical Hysterectomy – Lp. CISH; Pelviscopic Classic Intrafascial Supracervical Hysterectomy – Pelv. CISH; Vaginal Hysterectomy – VH; Intrafascial Vaginal Hysterectomy – IVH; Laparoscopic Assisted Vaginal Hysterectomy – LAVH.

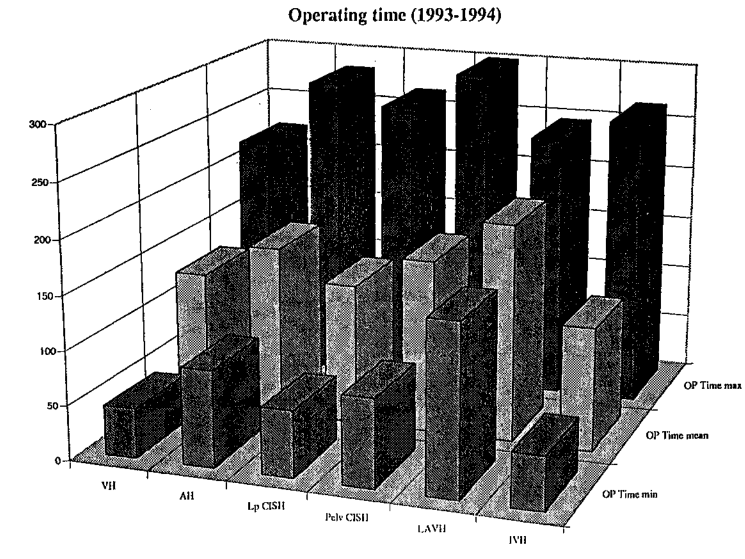

FIGURE 6 Operating time of 368 hysterectomies of patients who underwent hysterectomy at the Department of Obstetrics and Gynaecology University of Kiel (1993–1994). Abdominal Hysterectomy – AH; Laparotomic Classic Intrafascial Supracervical Hysterectomy – Lp. CISH; Pelviscopic Classic Intrafascial Supracervical Hysterectomy – Pelv. CISH; Vaginal Hysterectomy – VH; Intrafascial Vaginal Hysterectomy – IVH; Laparoscopic Assisted Vaginal Hysterectomy – LAVH.

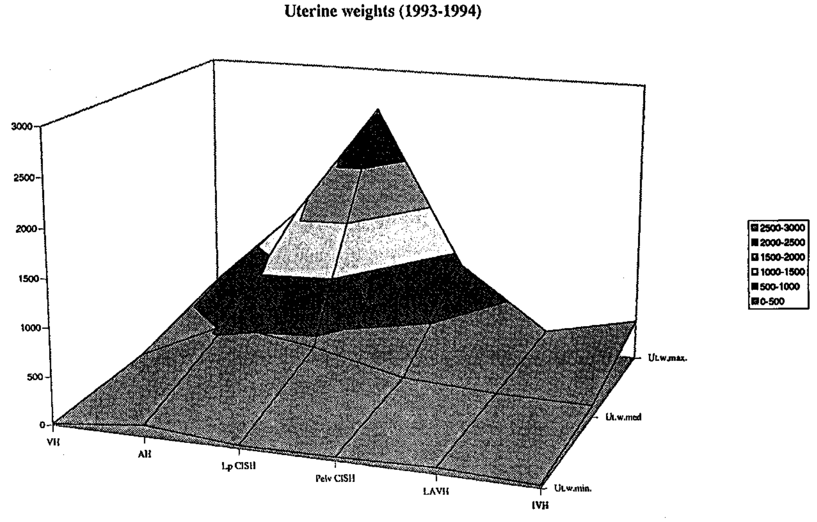

FIGURE 7 Uterine weights of 368 hysterectomies of patients who underwent hysterectomy at the Department of Obstetrics and Gynaecology University of Kiel (1993–1994). Abdominal Hysterectomy – AH; Laparotomic Classic Intrafascial Supracervical Hysterectomy – Lp. CISH; Pelviscopic Classic Intrafascial Supracervical Hysterectomy – Pelv. CISH; Vaginal Hysterectomy – VH; Intrafascial Vaginal Hysterectomy – IVH; Laparoscopic Assisted Vaginal Hysterectomy – LAVH.

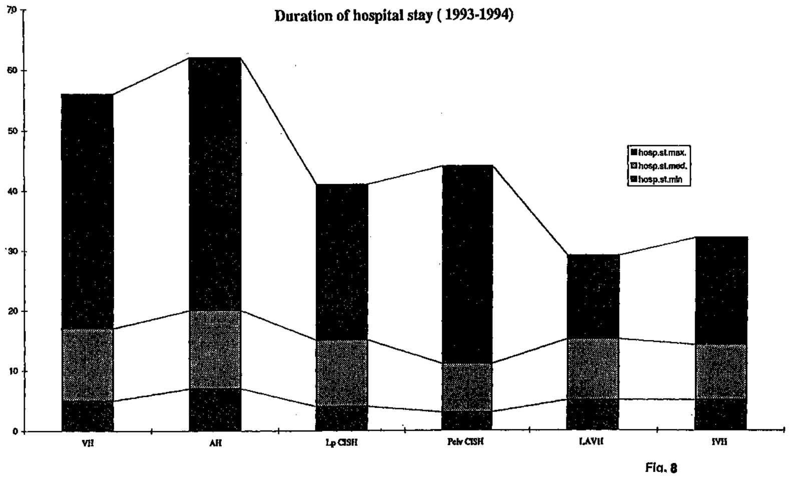

FIGURE 8 Durations of hospital stay (n=days) inpatients who underwent hysterectomies with benign indications in respect to different techniques of hysterectomy. Abdominal hysterectomy – AH; Laparotomic Classic Intrafascial Supracervical Hysterectomy – Lp. CISH; Pelviscopic Classic Intrafascial Supracervical Hysterectomy – Pelv. CISH; Vaginal hysterectomy – VH; Intrafascial vaginal hysterectomy– IVH; Laparoscopic Assisted Vaginal Hysterectomy – LAVH.

The shortest period of postoperative hospitalization was found among the patients, undergoing pelviscopic CISH operations (Fig. 8). The German medical insurance system allows all patients to stay in the hospital till they feel well – that explains the rather long hospital stay compared to other countries. The analgesics applied postoperatively did not show any statistically significant difference in any surgical procedures.

Only in four patients (1.08%) of pelvic surgery group was laparotomy needed because of technical difficulties or uncontrolled bleeding from the uterine pedicle. These cases were included in the laparotomy CISH group. Three patients had a postoperative stump infection confirmed by ultrasound and diagnostic laparoscopy. One of the cases needed pelviscopic drainage while the remaining two cases were treated with antibiotics. Four patients of CISH group returned to the clinic few days following the operations with secondary haemorrhage from the cervical stump. They were treated as outpatients with endocoagulation of the bleeding points and vaginal tamponade.

### DISCUSSION

This paper describes the impact of a new method of hysterectomy (CISH) on the pattern of hysterectomies performed at the University of Kiel. The advantages of this procedure are that CISH is associated with less blood-loss and a shorter hospital stay than other procedures.

The supracervical approach does not require the formal anatomic location of the ureters and the uterine arteries and should result in less disturbance to the rectum, bladder and vaginal function postoperatively. It may also be associated with better preservation of sexual feelings in the vaginal and cervical stump. These results were obtained by at least 15 surgeons and assistants and it is possible that a fairly long training period is required to obtain the best results from pelviscopic procedure.

After the initial publication of Semm in 1984[10] on laparoscopic adnexal devision to facilitate the difficult cases of vaginal hysterectomy, Reich in 1989 described total laparoscopic hysterectomy as a technique [11] in benign cases. Total laparoscopic hysterectomy was also reported in malignant cases by Dargent [13], Querleu [14] and Childers [15]. The variety of endoscopic hysterectomies available today open the door for a revolution in the old techniques.

Since the first hysterectomy was performed by Soranus from Ephesus (Greece) in a gangrenous uterus [16], the indication for each approach to hysterectomy continue to be a matter of controversy. From our data the most common indications for hysterectomies are fibroid uterus in cases of abdominal hysterectomy (62.8%), laparotomy CISH (73.3%), pelviscopic CISH (44.9%) and IVH (46%). Uterine prolapse is the most common indication for vaginal hysterectomy (61.36%) and IVH (22%). Menometrorrhagia was indicated for pelviscopic CISH in 33%. In spite of this fact the histological features showed no differences between laparotomy, pelviscopic CISH procedure and abdominal hysterectomy. The number of specimens without documentation of any pathology, range from 12.9% (pelviscopic CISH) to 36.4% (vaginal hysterectomy). These numbers are confirmed by other authors observations ranging om 4 to 38% [17,18]. It is interesting to note, that the mean operating time for our pelviscopic CISH procedure (158.99 min) was shorter than reported by other experienced surgeons: 210 minutes [19,20] and surprisingly similar to other types of pelviscopic hysterectomies: 160, 164 min for LAVH [21,22].

With our surgical experience in the CISH procedure we shall certainly be able to shorten the operating time.

### *References*

[1] Freund, W.A. Bemerkungen zu meiner Methode der uterus-extirpation. *Zent bl Gynäkol.* 1878; **2**: 497–500.

[2] Tervilä, I. Carcinoma of the cervical stump. *Acta Obstet. Gynecol. Scand.* 1963; **42**: 200.

[3] Mettler, L, Alvarez-Rodas, E., Lehmann-Willenbrock, L., Lüttges, E.J. and Semm, K. Intrafascial supracervical hysterectomy without colpotomy and transuterine mucosal resection by pelviscopy and laparotomy. *Diagn. Therap. Endosc.* 1995; **1**: 201–207.

[4] Mettler, L., Semm, K., Lütges, J.-E. and Panadicar, D. Pelviskopische intrafasciale Hysterectomy ohne Kolpotomy (CISH). *Gynäkol Prax.* 1993; **17**: 509–526.

[5] Mettler, L., Semm, K., Shah, A. and Shah, P. Intrafascial supracervical hysterectomy without colpotomy and transuterine mucosal resection by pelvis copy and laparotomy – our first 200 cases. *Current Investigation in Gynecol. and Obstet.* 1993: **9**: 349–362.

[6] Mettler, L., Semm, K., Lehmann-Willenbrock, L., Shah, A., Shah, P. and Sharma, R. Comparative evaluation of classical intrafascial-supracervical hysterectomy (CISH) with transuterine mucosal resection as performed by pelvis copy and laparotomy – our first 200 cases. *Surg. Endosc.* 1995; **9**: 418–423.

[7] Semm, K. Hysterektomie per laparotomiam oder per pelviscopiam. *Geburtsh. u Frauenheik.* 1991; **51**: 996–1003.

[8] Semm, K. Morzellieren und Nähen per pelviskopam-kein Problem mehr. *Geburtshh. u. Frauenheik.* 1991; **51**: 843–846.

[9] Semm, K. Prophylaxe der Sterilität durch minimal invasive Chirurgie. *Der Frauenarzt.* 1994; **35**: 944–954.

[10] Semm, K. Operationslehre für endoscopische Abdominal Chirurgie-operative Pelviscopie. Schattauer Verlag Stuttgart, New York. 1984. English translation, Year Book Medical Publishers Inc., Chicago, 1987.

[11] Reich, H., DeCaprio, J. and McGlyn, F. Laparoscopic hysterectomy. *J. Gynecol. Surg.* 1989; **5**: 213–216.

[12] Stoeckel, W. Zur Technik der vaginalen geburtshilflichen Untersuchung. *Zbl. Gynecol.* 1916, **39**: 161.

[13] Dargent, D. and Mathevet, P. Hysterectomie elargie laparoskopico-vaginale. *J. Gynecol. Obstet. Biol. Reprod. Paris* 1992; **21**: 709–710.

[14] Querleu, D. Laparoscopically assisted radical vaginal hysterectomy. *Gynecol-Oncol.* 1993; **51(2)**: 248–254.

[15] Childers, J.M., Hatch, K. and Surwit, E.A. The role of laparoscopic lymphadenectomy in the management of cervical carcinoma. *Gynecol-Oncol.* 1992, **47**: 38–43.

[16] Bachman, G.A. Hysterectomy. *J. Reprod. Med.* 1990; **35**: 839–862.

[17] Alvarez-Rodas, E., Mettler, L., Castro, E., Lüttges, E.J. and Semm, K. Histologic features of the CISH procedure. *J. Am. Assoc. Gynecol. Laparosc.* 1994; **2**: 37–41.

[18] Ojeda, V.J. The pathology of hysterectomy specimens. *NZ. Med. J.* 1979; **89**: 169.

[19] Vietz, P.F. and Ahn, T.S. A new approach to hysterectomy without colpotomy: pelviscopic intrafascial hysterectomy. *Am. J. Obstet. Gynecol.* 1994; **170**: 609–613.

[20] Vietz, P.F. and Ahn, T.S. From laparotomy to pelviscopic intrafascial hysterectomy. 1995. *In press.*

[21] Graidner, L., Bowen, L. and Delmore, J.E. Laparoscopic assisted vaginal hysterectomy: 50 consecutive cases compared to the traditional vaginal approach. *Fert. Ster.* 1992; **56**: 25.

[22] Nezhat, F., Nezhat, C., Gordon, S. and Wilkins, E. Laparoscopic versus abdominal hysterectomy. *J. Reprod. Med.* 1992; **37**: 247–250.

